# Selective deletion of endothelial mineralocorticoid receptor protects from vascular dysfunction in sodium-restricted female mice

**DOI:** 10.1186/s13293-020-00340-5

**Published:** 2020-11-23

**Authors:** Jessica L. Faulkner, Emily Lluch, Simone Kennard, Galina Antonova, Iris Z. Jaffe, Eric J. Belin de Chantemèle

**Affiliations:** 1 Vascular Biology Center, Medical College of Georgia at Augusta University, 1460 Laney Walker Blvd, Augusta, GA 30912 USA; 2 Molecular Cardiology Research Institute, Tufts Medical Center, Boston, MA USA; 3 Department of Cardiology, Medical College of Georgia at Augusta University, Augusta, GA USA

**Keywords:** Sex-differences, Aldosterone, Mineralocorticoid receptor, CYP11B2, Endothelial function, Nitric oxide, Vascular function, NOX4

## Abstract

**Background:**

Recent evidence by our laboratory demonstrates that women and female mice endogenously express higher endothelial mineralocorticoid receptor (ECMR) than males. Mounting clinical evidence also indicates that aldosterone production is higher in pathological conditions in females compared to males. However, the role for increased activation of ECMR by aldosterone in the absence of a comorbid condition is yet to be explored. The current study hypothesized that increased ECMR activation induced by elevated aldosterone production predisposes healthy female mice to endothelial dysfunction.

**Method:**

Vascular reactivity was assessed in aortic rings from wild-type (WT) and ECMR KO (KO) mice fed either a normal salt (NSD, 0.4% NaCl) or sodium-restricted diet (SRD, 0.05% NaCl) for 28 days.

**Results:**

SRD elevated plasma aldosterone levels as well as adrenal *CYP11B2* and angiotensin II type 1 receptor (AT1R) expressions in female, but not male, WT mice. In baseline conditions (NSD), endothelial function, assessed by vascular relaxation to acetylcholine, was higher while vascular contractility to phenylephrine, serotonin, and KCl lower in female than male WT mice. SRD impaired endothelial function and increased vascular contractility in female, but not male, WT mice effectively ablating the baseline sex differences. NOS inhibition with LNAME ablated endothelial relaxation to a higher extent in male than female mice on NSD and ablated differences in acetylcholine relaxation responses between NSD- and SRD-fed females, indicating a role for NO in SRD-mediated endothelial function. In association, SRD significantly reduced vascular NOX4 expression in female, but not male, mice. Lastly, selective deletion of ECMR protected female mice from SRD-mediated endothelial dysfunction and increased vascular contractility.

**Conclusion:**

Collectively, these data indicate that female mice develop aldosterone-induced endothelial dysfunction via endothelial MR-mediated reductions in NO bioavailability. In addition, these data support a role for ECMR to promote vascular contractility in female mice in response to sodium restriction.

## Background

Accumulating clinical evidence suggests that mineralocorticoid receptor (MR) antagonists provide a sex-specific benefit to cardiovascular health in women [1–6]. These clinical findings are likely attributable to an endogenous heightened vascular sensitivity to aldosterone-MR activation in females. More specifically, emerging experimental data indicate that the endothelial-specific MR (ECMR) is a critical mediator of vascular dysfunction in female animal models. In support of this notion, our lab recently published that endothelial MR expression is endogenously higher in female mice and humans compared to males [7]. Furthermore, we and others have demonstrated that ECMR deletion is protective against obesity-associated endothelial dysfunction and vascular stiffness [7–9]. However, these studies were unable to isolate the effects of ECMR activation on vascular function in the absence of other comorbidities associated with models of obesity.

In addition to an increased proclivity for endothelial MR expression, clinical and experimental data demonstrate that production of aldosterone is higher in females in response to pathological stimuli [10–12]. In a previous report, we demonstrated that inappropriately high aldosterone levels in salt-sensitive hypertensive female mice were associated with an increased expression of adrenal aldosterone synthase, the rate-limiting enzyme of aldosterone secretion (referred to as *CYP11B2*, the encoding gene whose nomenclature is commonly used interchangeably), as well as dysregulation of the localized adrenal renin-angiotensin system [13]. These data indicate that the sex-specific clinical benefits of MR antagonists on cardiovascular health in women are attributable to a 2-pronged sex difference of heightened ECMR expression and aldosterone production in women. To explore the sex-specific functional mechanisms via which aldosterone production increases as well as the vascular-centric functional mechanisms of heightened ECMR activation in females, we developed a model of aldosterone stimulation in the absence of a comorbid condition: the sodium restriction model.

## Methods

### Animal models

All procedures and protocols were approved by the Augusta University Institutional Animal Care and Use Committee (IACUC protocol #2011-0108) and are compliant with guidelines set forth by the NIH. All animals were housed in an American Association of Laboratory Animal Care-approved animal care facility at Augusta University. Animals were housed at ambient temperature with 12:12 h light-dark cycles with food and water ad libitum. Male and female littermate mice at 10 weeks of age with intact endothelial MR (WT) or endothelial-specific MR deletion (KO) originally provided by Dr. Iris Jaffe [14] (Tufts University) were bred and housed at Augusta University. KO mice (C57BL6 background) were originally generated by flanking loxP sites on MR gene exons in mice and breeding these with vascular endothelial (VE)-cadherin Cre recombinase transgene (Cre+) mice to achieve an endothelial cell-specific MR deletion. Breeding across generations continued with Cre^+^ heterozygotes bred with Cre^−^ homozygote mice, both with homozygote MR flox/flox, generating an even distribution of KO mice and WT littermates. Mice were fed either normal salt (0.4% NaCl, NSD) or sodium-restricted diet (Envigo cat#TD.160502, 0.05% NaCl, SRD) for 28 days. Mice were euthanized via decapitation under isoflurane anesthesia.

### Assessment of vascular reactivity

Excised thoracic aortas were cleaned of adipose tissue, cut in 2 mm rings, and mounted on DMT® wire myograph (Ann Arbor, MI). Concentration-response curves to phenylephrine, serotonin, sodium nitroprusside, and acetylcholine in the presence or absence of the nitric oxide synthase (NOS) inhibitor Nω-Nitro-L-arginine methyl ester hydrochloride (LNAME, 100 μM, 20 min preincubation) as well as maximum responses to KCl were performed and recorded with the LabChart® analysis software (AD Instruments®, Colorado Springs, CO) as previously described (1 nM-30 μm concentrations) [7, 13, 15, 16].

### Western blotting and quantitative real-time RT-PCR

Adrenal glands and aorta were homogenized, protein measured by bicinchoninic assay (BCA, ThermoFisher®, Waltham, MA), and then run on SDS-PAGE gels. Proteins were transferred to nitrocellulose membranes and stained for *CYP11B2* (aldosterone synthase, antibodies generously supplied by Drs. Elise and Celso Gomez-Sanchez, University of Mississippi Medical Center) and β-actin as previously described [7, 13]. In addition, RNA was isolated (Qiagen® minikit, Germantown, MD) from homogenates in Trizol (ThermoFisher®, Waltham, MA) followed by reverse transcription to obtain cDNA (ThermoFisher®, Waltham, MA). qRT-PCR was performed utilizing Sybr Green (Applied Biosystems®, Foster City, CA) and the primer sequences in Table 1. Ct values from each sample were normalized to 18 s expression within the sample (ΔCt) followed by normalization to control groups (ΔΔCt) prior to calculation of relative gene expression (2^−ΔΔCt^) as previously described [7, 13] and in accordance with Applied Biosystems guidelines (Applied Biosystems® User Bulletin, no. 2, 1997). 
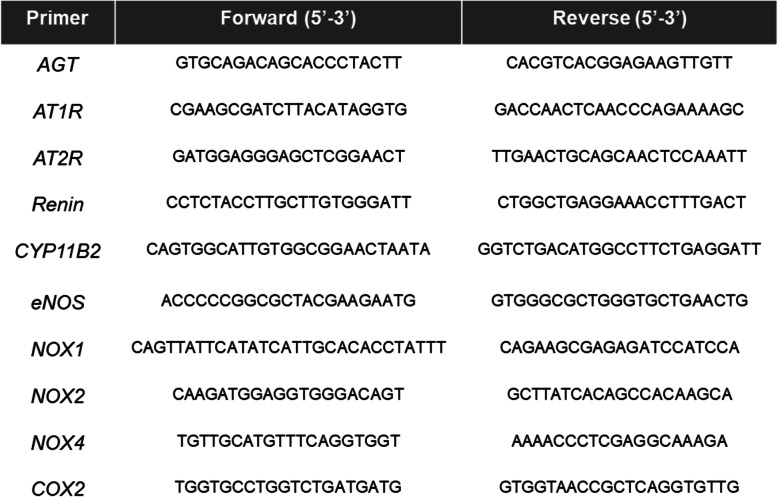

**Table 1:** Primer sequences for qRT-PCR

Primer	Forward (5’-3’)	Reverse (5’-3’)
*AGT*	GTGCAGACAGCACCCTACTT	CACGTCACGGAGAAGTTGTT
*AT1R*	CGAAGCGATCTTACATAGGTG	GACCAACTCAACCCAGAAAAGC
*AT2R*	GATGGAGGGAGCTCGGAACT	TTGAACTGCAGCAACTCCAAATT
*Renin*	CCTCTACCTTGCTTGTGGGATT	CTGGCTGAGGAAACCTTTGACT
*CYP11B2*	CAGTGGCATTGTGGCGGAACTAATA	GGTCTGACATGGCCTTCTGAGGATT
*eNOS*	ACCCCCGGCGCTACGAAGAATG	GTGGGCGCTGGGTGCTGAACTG
*NOX1*	CAGTTATTCATATCATTGCACACCTATTT	CAGAAGCGAGAGATCCATCCA
*NOX2*	CAAGATGGAGGTGGGACAGT	GCTTATCACAGCCACAAGCA
*NOX4*	TGTTGCATGTTTCAGGTGGT	AAAACCCTCGAGGCAAAGA
*COX2*	TGGTGCCTGGTCTGATGATG	GTGGTAACCGCTCAGGTGTTG

### Plasma aldosterone and electrolytes

Plasmas were collected at sacrifice (animals sacrificed prior to noon) in heparin saline. Plasma concentrations of aldosterone (Caymen Chemical®, Ann Arbor, MI) were measured utilizing commercially available assay (Detection range, 15.6-4000 pg/ml) as previously described [13]. Plasma Na^+^ and K^+^ levels were measured via PinAAcle 500 atomic absorption analyzer (Perkin Elmer, Waltham, MA).

### Statistical analysis

Statistical analysis was performed in the Graphpad Prism® software (La Jolla, CA). All bar data were expressed as mean ± S.E.M. In non-repeated variables, effects of diet and sex or diet, sex, and KO were measured by two-way ANOVA or three-way ANOVA, respectively, and differences among means measured by Sidak’s multiple comparison test. Analysis of dose-response curves utilized two-way ANOVA with repeated variable measures. *P* value of < 0.05 was considered significant.

## Results

### Sodium restriction does not alter body, heart, or kidney weight in male or female mice

Characterization of the general profile of the mice revealed that female mice have a significantly lower body weight than males which was neither affected by SRD nor ECMR deletion (Table 2). No significant differences were observed in kidney or heart weights as well as plasma Na^+^ or K^+^ levels regardless of sex, diet, or ECMR deletion. However, in response to SRD female mice demonstrated a lower uterine mass compared to NSD animals. 
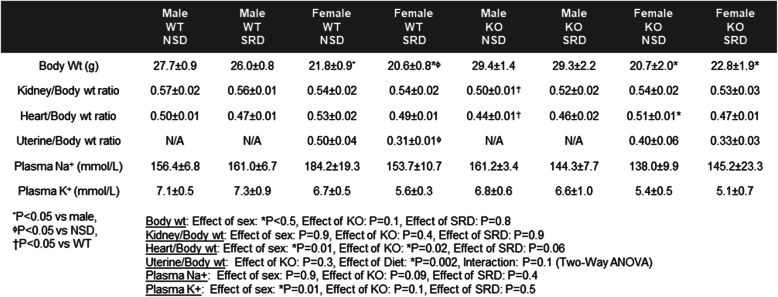

**Table 2:** Body, kidney, heart weight, and plasma Na^+^ and K^+^ in experimental groups. Measures of body weight, kidney, and heart weights expressed as a ratio to body weight as well as plasma sodium and potassium levels at sacrifice in male and female WT and KO mice on NSD or SRD. Three-way ANOVA with Sidak’s multiple comparisons test (*N* = 4-12)

	Male	Male	Female	Female	Male	Male	Female	Female
	WT	WT	WT	WT	KO	KO	KO	KO
	NSD	SRD	NSD	SRD	NSD	SRD	NSD	SRD
Body Wt (g)	27.7±0.9	26.0±0.8	21.8±0.9^*^	20.6±0.8*^ɸ^	29.4±1.4	29.3±2.2	20.7±2.0*	22.8±1.9*
Kidney/Body wt ratio	0.57±0.02	0.56±0.01	0.54±0.02	0.54±0.02	0.50±0.01^†^	0.52±0.02	0.54±0.02	0.53±0.03
Heart/Body wt ratio	0.50±0.01	0.47±0.01	0.53±0.02	0.49±0.01	0.44±0.01^†^	0.46±0.02	0.51±0.01*	0.47±0.01
Uterine/Body wt ratio	N/A	N/A	0.50±0.04	0.31±0.01^ϕ^	N/A	N/A	0.40±0.06	0.33±0.03
Plasma Na^+^ (mmol/L)	156.4±6.8	161.0±6.7	184.2±19.3	153.7±10.7	161.2±3.4	144.3±7.7	138.0±9.9	145.2±23.3
Plasma K^+^ (mmol/L)	7.1±0.5	7.3±0.9	6.7±0.5	5.6±0.3	6.8±0.6	6.6±1.0	5.4±0.5	5.1±0.7

^*^P < 0.05 vs male, ^ϕ^*P* < 0.05 vs NSD

^†^*P* < 0.05 vs WT

Body wt: Effect of sex: *P < 0.5, Effect of KO: *P* = 0.1, Effect of SRD: *P* = 0.8

Kidney/Body wt: Effect of sex: *P* = 0.9, Effect of KO: *P* = 0.4, Effect of SRD: *P* = 0.9

Heart/Body wt: Effect of sex: *P = 0.01, Effect of KO: *P = 0.02, Effect of SRD: *P* = 0.06

Uterine/Body wt: Effect of KO: *P* = 0.3, Effect of Diet: *P = 0.002, Interaction: *P* = 0.1 (Two-Way ANOVA)

Plasma Na+: Effect of sex: *P* = 0.9, Effect of KO: *P* = 0.09, Effect of SRD: *P* = 0.4

Plasma K+: Effect of sex: *P = 0.01, Effect of KO: *P* = 0.1, Effect of SRD: *P* = 0.5

### Sodium restriction increases plasma aldosterone levels and adrenal *CYP11B2* expression in female, but not male, mice

**Fig. 1:**
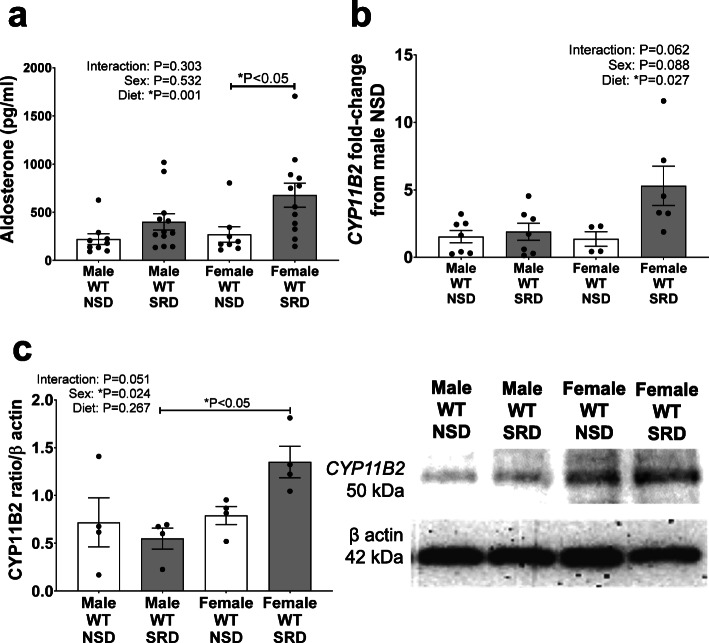
Sodium restriction increases aldosterone production and *CYP11B2* expression only in female mice. Plasma aldosterone levels (**a**), and adrenal mRNA (**b**), and protein expression (**c**) of *CYP11B2* in male and female mice on NSD and SRD. Two-way ANOVA with Sidak’s multiple comparisons test (*P < 0.05, *N* = 4-12)

Male and female mice demonstrated similar baseline aldosterone levels. Sodium restriction significantly increased plasma aldosterone levels independent of sex; however, post-hoc analysis demonstrated that SRD induced a significant increase in aldosterone levels only in female mice (Fig. 1a). Female sex and SRD trended to increase CYP11B2 mRNA (Fig. 1b), and significantly increased CYP11B2 protein (Fig. 1c), expressions. In addition, post-hoc analysis revealed that CYP11B2 protein expression was significantly higher in female compared to male SRD-fed mice. Collectively, these data indicate that SRD more potently increases aldosterone production in female than male mice.

### Sodium restriction increases adrenal angiotensin II type 1 receptor (AT1R) in female mice only

**Fig. 2:**
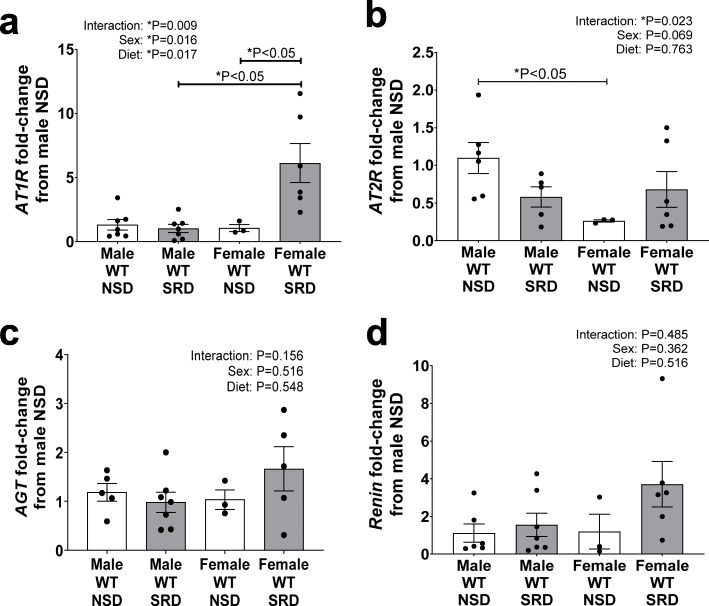
Adrenal *AT1R* expression increases with sodium restriction only in female mice. Adrenal mRNA expression of angiotensin II type I receptor (*AT1R*) (**a**), *AT2R* (**b**), angiotensinogen (*AGT*) (**c**), and *renin* (**d**) in male and female mice on NSD and SRD. Two-way ANOVA with Sidak’s multiple comparisons test (*P < 0.05, *N* = 3-7)

To investigate the potential mechanisms whether SRD increases adrenal aldosterone production sex specificity, we focused on intra-adrenal RAS activation. We measured adrenal angiotensin II types 1 and 2 receptors (AT1R, AT2R), angiotensinogen (AGT), and renin mRNA expression levels. The interaction of female sex and SRD significantly increased AT1R mRNA expression in adrenals of female mice and, in addition, post-hoc analysis revealed that SRD increased AT1R expression compared to both male SRD-fed and female NSD-fed mice (Fig. 2a). Moreover, female sex reduced adrenal AT2R expression in adrenal glands of mice on NSD, however, SRD ablated this sex difference (Fig. 2b). However, neither female sex nor SRD increased adrenal AGT or renin mRNA expressions (Fig. 2c, d). These data present SRD-associated heightened angiotensin II receptors expression as a potential candidate mechanism for increased adrenal aldosterone production in female mice.

### Sodium restriction impairs endothelial function in female mice only and ablates sex differences in endothelial function

**Fig. 3:**
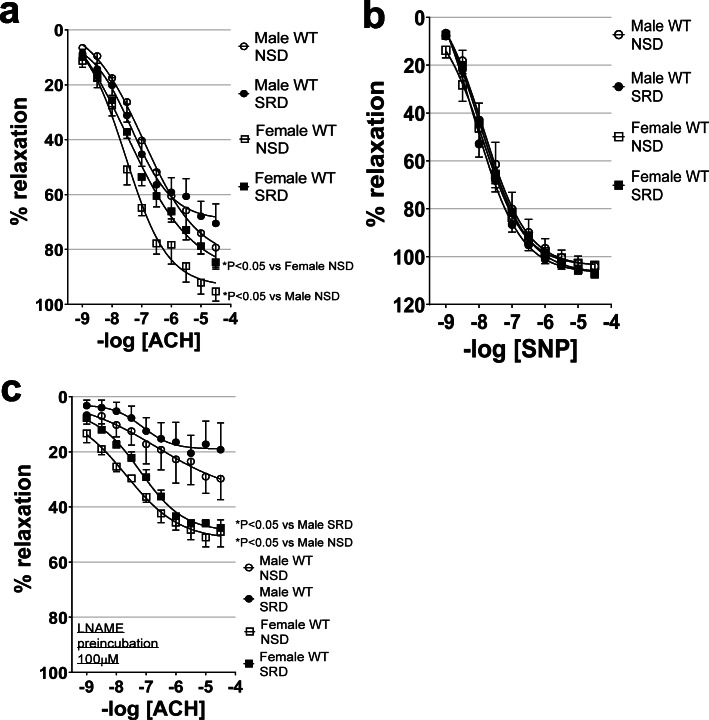
Sodium restriction impairs endothelial function in female mice only in an NO-dependent manner. Endothelial-dependent relaxation responses to acetylcholine (ACH) (**a**), endothelial-independent relaxation responses to sodium nitroprusside (SNP) (**b**), and relaxation responses to acetylcholine in the presence of nitric oxide synthase inhibitor Nω-Nitro-L-arginine methyl ester hydrochloride (LNAME) (**c**) in male and female mice on NSD or SRD. Two-way ANOVA of dose-response curves with repeated measures (*P < 0.05, male NSD *N* = 5, male SRD *N* = 8, female NSD *N* = 5, female SRD *N* = 11)

Endothelium-mediated relaxation, as assessed by vascular relaxation to acetylcholine, was endogenously higher in female than male mice on NSD (Fig. 3a). However, SRD impaired endothelial function in female, but not male mice, and effectively ablated the sex difference in relaxation to acetylcholine. Neither sex nor SRD impaired endothelial-independent relaxation to sodium nitroprusside in male and female mice, indicating that reductions in acetylcholine-mediated responses associated with male sex and SRD are due to impaired endothelial function (Fig. 3b).

### SRD-mediated endothelial dysfunction involves reduced NO bioavailability in female mice

Relaxation responses to acetylcholine were performed in the presence of the NOS-inhibitor LNAME to determine the potential role of NO bioavailability in sex- and SRD-mediated differences in endothelial function. LNAME induced a higher reduction in relaxation in males compared to females indicating that baseline endothelial-dependent relaxation in males is proportionately more NO-mediated than in females (Fig. 3c). LNAME significantly reduced vascular relaxation in both males and females and abolished the difference between NSD and SRD in female mice, indicating that SRD impairs acetylcholine-mediated relaxation via lowering NO bioavailability.

### Sodium restriction reduces vascular *eNOS* expression in both sexes but *NOX4* in female mice only

**Fig. 4:**
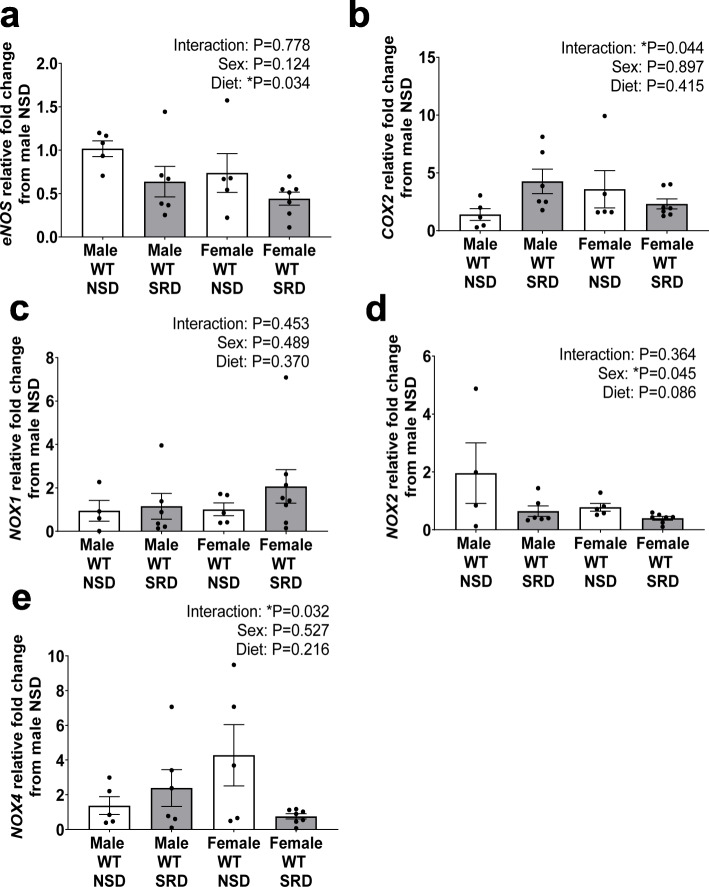
Sodium restriction decreases vascular *COX2* and *NOX4* expression in female mice. Vascular abdominal aorta mRNA expression of endothelial nitric oxide synthase (*eNOS*) (**a**), cyclooxygenase 2 (*COX2*) (**b**), nicotinamide adenine dinucleotide phosphate oxidase 1 (*NOX1*) (**c**), *NOX2* (**d**), and *NOX4* in male and female mice on NSD or SRD. Two-way ANOVA with Sidak’s multiple comparisons test (*P < 0.05, *N* = 4-7)

To identify potential mechanisms via which SRD decreases vascular NO bioavailability in female mice, we measured mRNA expressions of *eNOS*, *COX2*, *and NOX1*, *2* and *4* in aorta tissues. Figure 4a depicts that in baseline conditions (NSD) female sex does not increase *eNOS* mRNA expression; however, SRD decreased *eNOS* expression in a sex-independent manner. Baseline vascular *COX2* expression did not increase with female sex alone; however, the interaction of SRD and female sex significantly decreased *COX2* expression (Fig. 4b). Neither female sex nor SRD increased vascular *NOX1* expression (Fig. 4c). Female sex decreased *NOX2* expression; however, SRD did not increase *NOX2* expression in female mice (Fig. 4d). Female sex similarly did not increase vascular *NOX4* expression; however, the interaction of female sex and SRD significantly reduced vascular *NOX4* expression (Fig. 4e).

### Sodium restriction ablates sex differences in vascular contractility responses

**Fig. 5:**
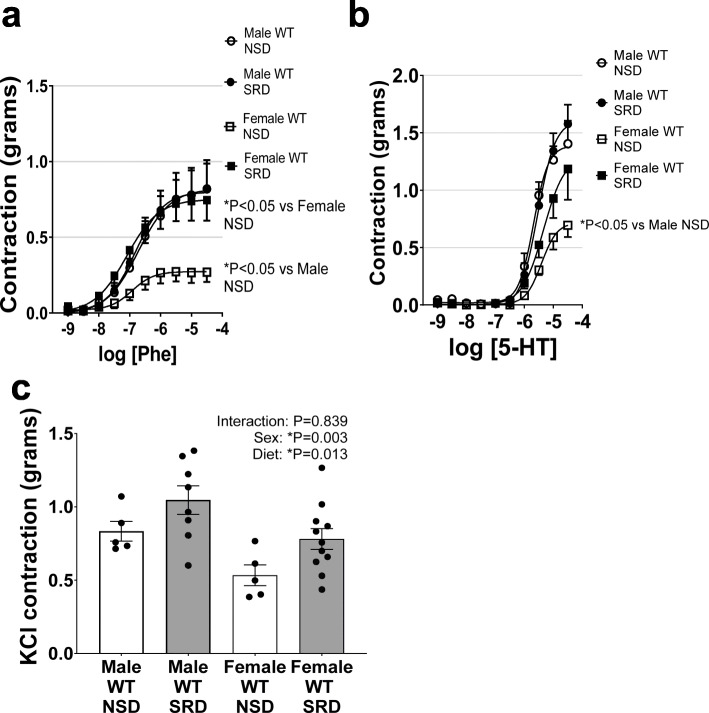
Sodium restriction ablates sex differences in vascular constriction responses by increasing constriction in female mice. Vascular constriction responses to phenylephrine (Phe) (**a**), serotonin (5-HT) (**b**), and KCl (**c**) in male and female mice on NSD or SRD. Two-way ANOVA of dose-response curves with repeated measures (*P < 0.05, male NSD *N* = 5, male SRD *N* = 8, female NSD *N* = 5, female SRD *N* = 11)

Female sex reduced vascular contractility as assessed by responses to the α-adrenergic receptor agonist phenylephrine, serotonin, and KCl (Fig. 5a-c). While sodium restriction had no effect to increase vascular contractility to phenylephrine and serotonin in male mice, SRD induced an increase in these responses in female mice, effectively ablating the baseline sex difference in contractility. Interestingly, although female sex reduced KCl-induced contractile responses, the statistical effect of SRD to increase KCl contraction was sex independent. Therefore, SRD increases vascular depolarization responses in a sex-independent manner, but female sex heightens sensitivity to SRD-mediated increases in α-adrenergic and serotonin receptor-derived vascular contractility.

### ECMR deletion protects female mice from SRD-mediated vascular dysfunction

**Fig. 6:**
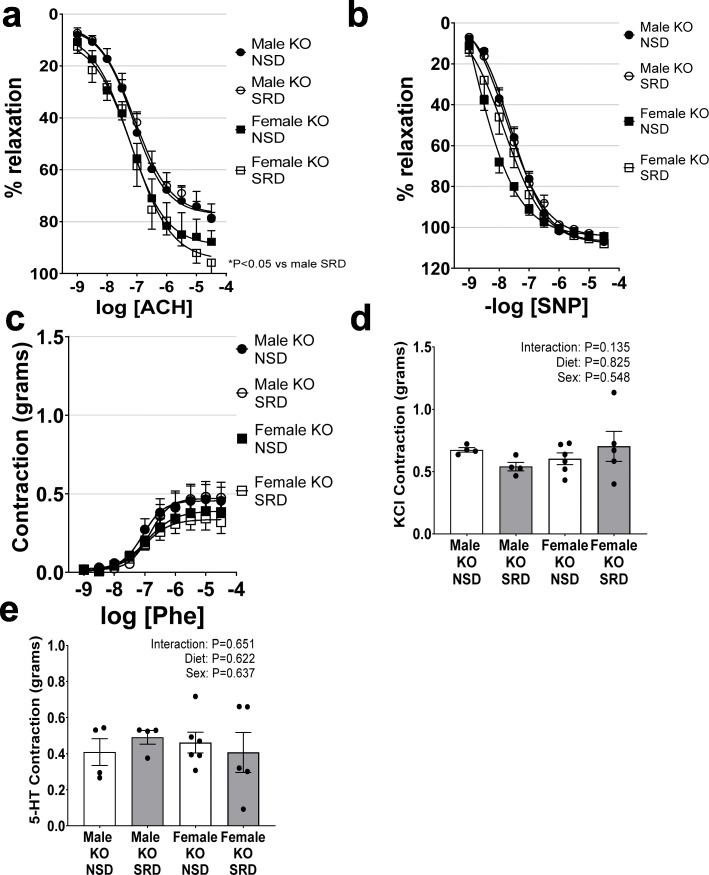
Endothelial MR deletion protects female mice from sodium restriction-induced endothelial dysfunction and vascular constriction. Endothelial relaxation responses to acetylcholine (Ach) (**a**), endothelial-independent relaxation responses to sodium nitroprusside (SNP) (**b**), vascular constriction responses to phenylephrine (Phe) (**c**), serotonin (5-HT) (**d**), and KCl (**e**) in male and female WT and KO mice on NSD and SRD. Two-way ANOVA of dose-response curves with repeated measures (*P < 0.05, male NSD KO *N* = 4, male KO SRD *N* = 4, female KO NSD *N* = 6, female KO SRD *N* = 5)

We and others have previously demonstrated an increased proclivity for ECMR activation in female mice [7, 17]; therefore, we utilized ECMR KO (KO) littermate male and female mice to determine whether SRD-mediated vascular dysfunction is ECMR mediated. In female mice with specific deletion of ECMR (KO), SRD failed to induce endothelial dysfunction as assessed by relaxation responses to acetylcholine (Fig. 6a), indicating a mediatory role for ECMR activation in SRD-induced endothelial impairment. Additionally, SRD had no effect on endothelial-independent relaxation responses to sodium nitroprusside in KO male or female mice (Fig. 6b). ECMR deletion also ablated the effect of male sex and SRD to increase vascular constriction to phenylephrine, serotonin, and KCl in mice (Fig. 6c-d).

### ECMR deletion restores SRD-mediated decreases in vascular *COX2* and *NOX4* expressions

**Fig. 7:**
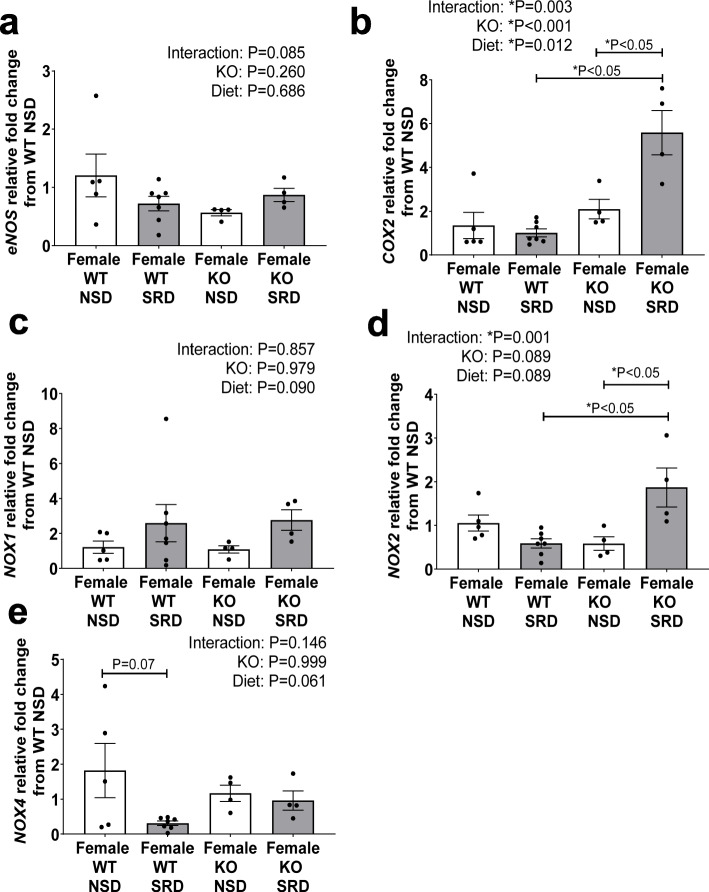
Endothelial MR deletion restores COX2 and NOX4 expression in female mice on SRD. Vascular abdominal aorta mRNA expression of endothelial nitric oxide synthase (*eNOS*) (**a**), cyclooxygenase 2 (*COX2*) (**b**), nicotinamide adenine dinucleotide phosphate oxidase 1 (*NOX1*) (**c**), *NOX2* (**d**), and *NOX4* in female WT and KO mice on NSD or SRD. Two-way ANOVA with Sidak’s multiple comparisons test (*P < 0.05, *N* = 4-7)

ECMR deletion (KO) did not increase vascular (aorta) *eNOS* expression at baseline or with SRD feeding (Fig. 7a). However, KO and SRD significantly increased vascular *COX2* expression and post-hoc analysis revealed an increased *COX2* expression in KO SRD-fed compared to both WT SRD-fed and KO NSD-fed females (Fig. 7b). Neither SRD nor KO increased *NOX1* expression (Fig. 7c). SRD significantly increased *NOX2* expression in KO females, and in addition, KO restored *NOX4* expressions to baseline levels in SRD-fed females (Fig. 7d, e).

## Discussion

In the current manuscript, we present data indicating that sodium restriction ablates vascular protective mechanisms that confer sex-specific higher endothelial function and lower vascular contraction in female mice via endothelial MR-mediated pathways. Our novel data indicates that (1) SRD sex specifically increases aldosterone production in association with an increase in adrenal *CYP11B2* and *ATR* expression, (2) sodium restriction impairs endothelial function in female mice, ablating the baseline sex difference, via reductions in NO bioavailability, (3) sodium restriction increases vascular contractility in female mice, ablating baseline sex differences, and (4) endothelial MR deletion (KO) protects female mice from SRD-induced endothelial dysfunction and increased vascular contractility. Collectively, these data indicate that increases in aldosterone production in otherwise healthy female mice increases sensitivity for vascular impairment via endothelial MR activation.

In association with clinical studies [10], our data indicate that aldosterone production elevates more acutely in female mice in response to the SRD stimulus. Previous reports indicate that the female sex hormone progesterone [18] increases aldosterone production while the male sex hormone testosterone [19–22] as well as aromatase inhibition [21, 23, 24] is associated with decreased aldosterone production. However, these regulatory pathways are scarcely understood at present. Notably, SRD concurrently increased adrenal *CYP11B2* expression only in female mice in the present report. In a previous study, we demonstrated that *CYP11B2* expression increases sex specifically in response to a high salt diet in female mice in association with heightened adrenal *AGT* expression but no change in *renin* or *AT1R* expression [13]. However, SRD, in contrast to the high salt diet in our previous study, did not increase adrenal *AGT* expression but did increase *AT1R* mRNA. Therefore, we presume that SRD-mediated increases in aldosterone production are likely mediated via increased ANGII activation of adrenal *AT1R*. This notion is supported by a recent clinical report which indicates that women produce more aldosterone in response to an ANGII stimulus than men [10]. A limitation of the current study is the lack of knowledge of whether ANGII levels were sex specifically increased by SRD, which warrants further investigation.

An important strength to our current data is that we utilized littermates, providing highly controlled baseline (NSD) conditions in which sex is the sole variable. We demonstrate a direct statistical comparison of the endothelial function in aortas of male and female mice on NSD, revealing a baseline sex difference favoring higher endothelial function in females, which correlates with clinical reports of higher flow-mediated dilation in healthy premenopausal women compared to men [25–28]. Despite the seeming protection favoring higher endothelial function in young women, emerging experimental and clinical data indicates that females are more susceptible to endothelial damage by pathological conditions. Previous studies show that diabetes [29], obesity [16, 17], and high salt diet [13], blunts acetylcholine relaxation responses more pronouncedly in female rodents than in males. These data are in line with clinical reports indicating that obese [30] and hypertensive [31] women present greater impairments in flow-mediated dilation than men. Given that the endothelium of females is more sensitive to damage and that endothelial dysfunction is a significant predictor for cardiovascular events [32, 33], the preservation of endothelial health is of particularly high clinical relevance in women.

Sex differences in both endothelial function and vascular contractility were effectively ablated with a sodium-restricted diet in our study, which was solely attributable to adverse effects on vascular relaxation and constriction in females. In addition, we observed a sex difference in the contribution of NO to endothelial relaxation in our WT littermate mice, favoring a lower contribution of NO to relaxation in females than in males. These data concur with data from others demonstrating that the contribution of other relaxation factors, notably endothelial-derived hyperpolarizing factor (EDHF), is higher in female mice and rats than in males in the mediation of baseline acetylcholine responses [17, 29]. In addition, our novel data demonstrate that SRD impairs endothelial function in female mice via reducing NO bioavailability, as relaxation responses in the presence of LNAME lost the SRD-mediated impairment effect. We also show that while vascular *eNOS* expression is decreased with SRD in a sex-independent manner, *COX2* is not specifically increased by female sex but decreases with SRD only in females. These data indicate that SRD induces a compensatory increase in *COX2* in males, and presumably increases vasodilatory prostaglandin production, but females lack this protective mechanism. This notion is supported by studies in humans [34, 35] and in eNOS-deficient mice [36, 37] which suggest that endothelial-dependent relaxation responses can be rescued by COX2-mediated prostaglandin production in disease states. In addition, we demonstrate that *NOX4* expression significantly decreases in female mice specifically on SRD. Endothelial NOX4 is a pleiotropic factor that diversely regulates oxidative stress pathways and NO bioavailability. In mice, deletion of NOX4 subsequently promotes endothelial dysfunction [38], and decreases NO production [39], which may be attributable to its well-noted ability to increase hydrogen peroxide and decrease reactive oxygen species formation [40]. Interestingly, a previous study demonstrated that aldosterone increases *NOX4* expression in cultured endothelial cells, however, this study did not differentiate by sex and indicates further that the decrease in *NOX4* by SRD in the current study warrants investigation of the female sex-specific regulatory mechanisms [41]. Therefore, baseline sex differences in endothelial function are likely mediated by increased EDHF contribution in females of this mouse strain, however, protection from SRD-mediated endothelial impairment in males may be attributable to protective effects of COX2- and NOX4-derived vasorelaxation.

Our data indicate that SRD-mediated increases in aldosterone production and higher endogenous ECMR expression are the key mediatory mechanisms for the loss of female sex-conferred protection from endothelial dysfunction and vascular contractility. Although aldosterone activates MR in numerous organ systems, we provide novel data indicating that SRD-induced aldosterone impairs vascular function via endothelial-specific MR activation in female mice, as deletion of this receptor in our KO mice ablated SRD effects to induce endothelial impairment and promote vascular contractility. Endothelial MR activation stimulates vascular inflammation and reactive oxygen species formation which are detrimental to NO bioavailability [42, 43]. Our data indicate that endothelial MR deletion also results in increases in vascular *COX2* and *NOX4* expressions in response to SRD in female mice. Collectively, these data indicate a novel role for endothelial MR to regulate COX2 and NOX4 in female mice, which may be key mediators of aldosterone-induced endothelial dysfunction and vascular contractility in mechanisms in females, warranting future investigation.

The data generated in this report utilized active cycling female mice and age-matched males, indicating these data pertain to premenopausal females. We further acknowledge an additional caveat in the study regarding that the mice were utilized at a young age, starting at 10 weeks at the initiation of SRD. Vascular function notably decreases with age and menopausal status in females, however, whether the sodium-restricted model results in vascular impairment in post-cycling age or ovariectomized mice warrants investigation [44]. This is particularly relevant given our data indicating that SRD reduced uterine weight, a crude measure of estrogen level, in female mice. These data indicate that SRD potentially interrupted natural estrous cycling patterns. The literature reports that the luteal phase of the menstrual cycle is associated with elevated plasma aldosterone level [18] and blood pressure [45]. Therefore, the SRD in the female mice may have served as a trigger to arrest in the diestrous (luteal in humans) phase of the estrous cycle, which is characterized by higher progesterone levels. Our previous report [7] demonstrated that progesterone increases endothelial MR expression; therefore, the female mice on SRD in the current study may have been sensitized to endothelial MR activation by a higher endothelial MR expression. In addition, future studies are warranted to determine whether these lower estrogen levels associated with the diestrous phase may contribute to the lower eNOS expression and vascular dysfunction in female mice on SRD and further whether these changes result in a change in blood pressure.

It is important to note that the current study does not advocate against “low salt diets,” whose cardiovascular benefits are well established [46]. The diet utilized in this study contains nearly no sodium at 0.05% which is a much lower sodium content than those recommended by the American Heart Association for humans and is a level of sodium content rarely consumed in a human’s diet. The experimental design of the current study represents a model of endogenous elevations in plasma aldosterone level by a physiological mechanism, the SRD. A previous study by our laboratory demonstrated that aldosterone is inappropriately increased in response to high salt diets in female mice, and the current study demonstrates that females are also more sensitive to sodium restriction-induced aldosterone production.

These studies also indicate the need for an important consideration for young hypertensive females on diuretics in that these therapies may have adverse effects on plasma aldosterone levels. The available literature on diuretic efficacy analyzed by both age and sex is limited at present; however, the SPRINT trial demonstrated that thiazide prescription prevalence may be higher in women than in men [47]. Both thiazide and loop diuretics increase sodium excretion and plasma aldosterone levels, indicating that they may be less efficacious in women than in men and aldosterone levels should be monitored as part of ongoing treatment with such agents in young women. In addition, the efficacy of MR antagonists vs other diuretics in hypertensive premenopausal women warrants clinical investigation.

## Perspectives and significance

Given the clinical data that MR antagonists are more efficacious for cardiovascular protection in women [1–6], these data indicate that dietary control of sodium levels, and subsequently control of aldosterone production and MR activation, is a crucial clinical target for women.

## Data Availability

The datasets used and/or analyzed during the current study are available from the corresponding author on reasonable request.

## References

[1] Chen J (2010). Sodium sensitivity of blood pressure in Chinese populations. Curr Hypertens Rep.

[2] Elliott P, Dyer A, Stamler R (1989). The INTERSALT study: results for 24 hour sodium and potassium, by age and sex. INTERSALT co-operative research group. J Hum Hypertens.

[3] Gwoo S, Kim YN, Shin HS, Jung YS, Rim H (2014). Predictors of hyperkalemia risk after hypertension control with aldosterone blockade according to the presence or absence of chronic kidney disease. Nephron Clin Pract.

[4] Kanashiro-Takeuchi RM, Heidecker B, Lamirault G, Dharamsi JW, Hare JM (2009). Sex-specific impact of aldosterone receptor antagonism on ventricular remodeling and gene expression after myocardial infarction. Clin Transl Sci.

[5] Michikawa T, Nishiwaki Y, Okamura T, Asakura K, Nakano M, Takebayashi T (2009). The taste of salt measured by a simple test and blood pressure in Japanese women and men. Hypertens Res.

[6] Olivieri O, Pizzolo F, Ciacciarelli A, Corrocher R, Signorelli D, Falcone S (2008). Menopause not aldosterone-to-renin ratio predicts blood pressure response to a mineralocorticoid receptor antagonist in primary care hypertensive patients. Am J Hypertens.

[7] Faulkner JL, Kennard S, Huby AC, Antonova G, Lu Q, Jaffe IZ (2019). Progesterone predisposes females to obesity-associated Leptin-mediated endothelial dysfunction via Upregulating endothelial MR (mineralocorticoid receptor) expression. Hypertension..

[8] Davel AP, Anwar IJ, Jaffe IZ (2017). The endothelial mineralocorticoid receptor: mediator of the switch from vascular health to disease. Curr Opin Nephrol Hypertens.

[9] Jia G, Habibi J, Aroor AR, Martinez-Lemus LA, DeMarco VG, Ramirez-Perez FI (2016). Endothelial mineralocorticoid receptor mediates diet-induced aortic stiffness in females. Circ Res.

[10] Shukri MZ, Tan JW, Manosroi W, Pojoga LH, Rivera A, Williams JS (2018). Biological sex modulates the adrenal and blood pressure responses to angiotensin II. Hypertension..

[11] Ahmed AH, Gordon RD, Taylor PJ, Ward G, Pimenta E, Stowasser M (2011). Are women more at risk of false-positive primary aldosteronism screening and unnecessary suppression testing than men?. J Clin Endocrinol Metab.

[12] Goodfriend TL, Kelley DE, Goodpaster BH, Winters SJ (1999). Visceral obesity and insulin resistance are associated with plasma aldosterone levels in women. Obes Res.

[13] Faulkner JL, Harwood D, Bender L, Shrestha L, Brands MW, Morwitzer MJ (2018). Lack of suppression of aldosterone production leads to salt-sensitive hypertension in female but not male balb/C mice. Hypertension..

[14] Mueller KB, Bender SB, Hong K, Yang Y, Aronovitz M, Jaisser F (2015). Endothelial mineralocorticoid receptors differentially contribute to coronary and mesenteric vascular function without modulating blood pressure. Hypertension..

[15] Huby AC, Antonova G, Groenendyk J, Gomez-Sanchez CE, Bollag WB, Filosa JA (2015). Adipocyte-derived hormone leptin is a direct regulator of aldosterone secretion, which promotes endothelial dysfunction and cardiac fibrosis. Circulation..

[16] Huby AC, Otvos L (2016). Belin de Chantemele EJ. Leptin induces hypertension and endothelial dysfunction via aldosterone-dependent mechanisms in obese female mice. Hypertension..

[17] Davel AP, Lu Q, Moss ME, Rao S, Anwar IJ, DuPont JJ (2018). Sex-specific mechanisms of resistance vessel endothelial dysfunction induced by cardiometabolic risk factors. J Am Heart Assoc.

[18] Szmuilowicz ED, Adler GK, Williams JS, Green DE, Yao TM, Hopkins PN (2006). Relationship between aldosterone and progesterone in the human menstrual cycle. J Clin Endocrinol Metab.

[19] More AS, Mishra JS, Hankins GD, Kumar S (2016). Prenatal testosterone exposure decreases aldosterone production but maintains normal plasma volume and increases blood pressure in adult female rats. Biol Reprod.

[20] Kau MM, Lo MJ, Wang SW, Tsai SC, Chen JJ, Chiao YC (1999). Inhibition of aldosterone production by testosterone in male rats. Metabolism..

[21] Toot J, Jenkins C, Dunphy G, Boehme S, Hart M, Milsted A (2008). Testosterone influences renal electrolyte excretion in SHR/y and WKY males. BMC Physiol.

[22] Johannsson G, Gibney J, Wolthers T, Leung KC, Ho KK (2005). Independent and combined effects of testosterone and growth hormone on extracellular water in hypopituitary men. J Clin Endocrinol Metab.

[23] Browne LJ, Gude C, Rodriguez H, Steele RE, Bhatnager A (1991). Fadrozole hydrochloride: a potent, selective, nonsteroidal inhibitor of aromatase for the treatment of estrogen-dependent disease. J Med Chem.

[24] Lipton A, Harvey HA, Demers LM, Hanagan JR, Mulagha MT, Kochak GM (1990). A phase I trial of CGS 16949A. A new aromatase inhibitor. Cancer..

[25] Skaug EA, Aspenes ST, Oldervoll L, Morkedal B, Vatten L, Wisloff U (2013). Age and gender differences of endothelial function in 4739 healthy adults: the HUNT3 fitness study. Eur J Prev Cardiol.

[26] Harris RA, Tedjasaputra V, Zhao J, Richardson RS (2012). Premenopausal women exhibit an inherent protection of endothelial function following a high-fat meal. Reprod Sci.

[27] Moreau KL, Hildreth KL, Klawitter J, Blatchford P, Kohrt WM. Decline in endothelial function across the menopause transition in healthy women is related to decreased estradiol and increased oxidative stress. Geroscience. 2020. 10.1007/s11357-020-00236-7. 10.1007/s11357-020-00236-7 PMC7732894 32770384

[28] Holder SM, Brislane A, Dawson EA, Hopkins ND, Hopman MTE, Cable NT (2019). Relationship between endothelial function and the eliciting shear stress stimulus in women: changes across the lifespan differ to men. J Am Heart Assoc.

[29] Zhang R, Thor D, Han X, Anderson L, Rahimian R (2012). Sex differences in mesenteric endothelial function of streptozotocin-induced diabetic rats: a shift in the relative importance of EDRFs. Am J Physiol Heart Circ Physiol.

[30] Suboc TM, Dharmashankar K, Wang J, Ying R, Couillard A, Tanner MJ (2013). Moderate obesity and endothelial dysfunction in humans: influence of gender and systemic inflammation. Physiol Rep.

[31] Cao C, Hu J, Dong Y, Zhan R, Li P, Su H (2015). Gender differences in the risk factors for endothelial dysfunction in Chinese hypertensive patients: homocysteine is an independent risk factor in females. PLoS One.

[32] Deanfield JE, Halcox JP, Rabelink TJ (2007). Endothelial function and dysfunction: testing and clinical relevance. Circulation..

[33] Villar IC, Francis S, Webb A, Hobbs AJ, Ahluwalia A (2006). Novel aspects of endothelium-dependent regulation of vascular tone. Kidney Int.

[34] Bulut D, Liaghat S, Hanefeld C, Koll R, Miebach T, Mugge A (2003). Selective cyclo-oxygenase-2 inhibition with parecoxib acutely impairs endothelium-dependent vasodilatation in patients with essential hypertension. J Hypertens.

[35] Szerafin T, Erdei N, Fulop T, Pasztor ET, Edes I, Koller A (2006). Increased cyclooxygenase-2 expression and prostaglandin-mediated dilation in coronary arterioles of patients with diabetes mellitus. Circ Res.

[36] Chataigneau T, Feletou M, Huang PL, Fishman MC, Duhault J, Vanhoutte PM (1999). Acetylcholine-induced relaxation in blood vessels from endothelial nitric oxide synthase knockout mice. Br J Pharmacol.

[37] Sun D, Huang A, Smith CJ, Stackpole CJ, Connetta JA, Shesely EG (1999). Enhanced release of prostaglandins contributes to flow-induced arteriolar dilation in eNOS knockout mice. Circ Res.

[38] Langbein H, Shahid A, Hofmann A, Mittag J, Bornstein SR, Morawietz H, et al. NADPH oxidase 4 mediates the protective effects of physical activity against obesity-induced vascular dysfunction. Cardiovasc Res. 2019. 10.1093/cvr/cvz322. 10.1093/cvr/cvz322 31800011

[39] Sanchez-Gomez FJ, Calvo E, Breton-Romero R, Fierro-Fernandez M, Anilkumar N, Shah AM (2015). NOX4-dependent hydrogen peroxide promotes shear stress-induced SHP2 sulfenylation and eNOS activation. Free Radic Biol Med.

[40] Rajaram RD, Dissard R, Jaquet V, de Seigneux S (2019). Potential benefits and harms of NADPH oxidase type 4 in the kidneys and cardiovascular system. Nephrol Dial Transplant.

[41] Caprio M, Newfell BG, la Sala A, Baur W, Fabbri A, Rosano G (2008). Functional mineralocorticoid receptors in human vascular endothelial cells regulate intercellular adhesion molecule-1 expression and promote leukocyte adhesion. Circ Res.

[42] Faulkner JL, Belin de Chantemele EJ (2019). Mineralocorticoid receptor and endothelial dysfunction in hypertension. Curr Hypertens Rep.

[43] Moss ME, Lu Q, Iyer SL, Engelbertsen D, Marzolla V, Caprio M (2019). Endothelial mineralocorticoid receptors contribute to vascular inflammation in atherosclerosis in a sex-specific manner. Arterioscler Thromb Vasc Biol.

[44] Rajasekar R, Andersson J, Leanderson T (1988). Heterogeneity in the specificities of an alloreactive T helper cell line. Scand J Immunol.

[45] Edwards N, Wilcox I, Polo OJ, Sullivan CE (1996). Hypercapnic blood pressure response is greater during the luteal phase of the menstrual cycle. J Appl Physiol (1985).

[46] Chiavaroli L, Viguiliouk E, Nishi SK, Blanco Mejia S, Rahelic D, Kahleova H (2019). DASH dietary pattern and cardiometabolic outcomes: an umbrella review of systematic reviews and meta-analyses. Nutrients..

[47] Chang TI, Evans G, Cheung AK, Cushman WC, Diamond MJ, Dwyer JP (2016). Patterns and correlates of baseline thiazide-type diuretic prescription in the systolic blood pressure intervention trial. Hypertension..

